# Behaviour of mesopredatory coral reef fishes in response to threats from sharks and humans

**DOI:** 10.1038/s41598-023-33415-5

**Published:** 2023-04-25

**Authors:** A. Asunsolo-Rivera, E. Lester, T. Langlois, B. Vaughan, M. I. McCormick, S. D. Simpson, M. G. Meekan

**Affiliations:** 1 School of Biological Sciences, The University of Western Australia Oceans Institute, University of Western Australia, Crawley, WA Australia; 2 Australian Institute of Marine Science, The University of Western Australia Oceans Institute, University of Western Australia, Crawley, WA Australia; 3 Coastal Marine Field Station, School of Science, University of Waikato, Tauranga, New Zealand; 4 School of Biological Sciences, University of Bristol, Bristol, UK

**Keywords:** Ecology, Ecology, Environmental sciences, Ocean sciences

## Abstract

Both sharks and humans present a potentially lethal threat to mesopredatory fishes in coral reef systems, with implications for both population dynamics and the role of mesopredatory fishes in reef ecosystems. This study quantifies the antipredator behaviours mesopredatory fishes exhibit towards the presence of large coral reef carnivores and compares these behavioural responses to those elicited by the presence of snorkelers. Here, we used snorkelers and animated life-size models of the blacktip reef shark (*Carcharhinus melanopterus*) to simulate potential predatory threats to mesopredatory reef fishes (lethrinids, lutjanids, haemulids and serranids). The responses of these reef fishes to the models and the snorkelers were compared to those generated by three non-threatening controls (life-size models of a green turtle [*Chelonia mydas*], a PVC-pipe [an object control] and a Perspex shape [a second object control]). A Remote Underwater Stereo-Video System (Stereo-RUV) recorded the approach of the different treatments and controls and allowed accurate measurement of Flight Initiation Distance (FID) and categorization of the type of flight response by fishes. We found that mesopredatory reef fishes had greater FIDs in response to the approach of threatening models (1402 ± 402–1533 ± 171 mm; mean ± SE) compared to the controls (706 ± 151–896 ± 8963 mm). There was no significant difference in FID of mesopredatory fishes between the shark model and the snorkeler, suggesting that these treatments provoked similar levels of predator avoidance behaviour. This has implications for researchers monitoring behaviour in situ or using underwater census as a technique to estimate the abundance of reef fishes. Our study suggests that, irrespective of the degree to which sharks actually consume these mesopredatory reef fishes, they still elicit a predictable and consistent antipredator response that has the potential to create risk effects.

## Introduction

Predators are a key determinant of the structure of ecosystems, impacting energy flows and nutrient cycling both through the consumption of prey and by inducing trait responses in prey species that mitigate predation risk^1–4^. These risk-induced trait responses include modifications in prey behaviour^5^ (referred to hereafter as antipredator behaviours). Predation avoidance behaviours can result in reduced access to food and mates and can ultimately influence the demography, fitness, growth and morphology of prey^6–8^. Such non-consumptive effects of predators are known as predation-risk effects^2,5,9^.

In both terrestrial and marine ecosystems, the guild of predators at or near the top of the food chain includes humans^10^. As humans are free from many of the energetic and physical constraints facing other nonhuman predators, they can exploit large-bodied mesopredator and carnivore guilds at very high rates, which has resulted in them being termed “super-predators”^11,12^. In terrestrial systems, where humans and large carnivores compete for the same prey, the presence of humans or human cues can have pervasive effects on these ecological communities. For example, the sound of human vocalisations can trigger avoidance responses in large carnivores and reduce foraging in medium-sized carnivores, which in turn benefits small mammals that increase foraging and habitat use^13^. In marine systems, where large predators are highly mobile^14^ and have been severely depleted^15^, the extent to which humans and large carnivores contribute to ecosystem function and ultimately the expression of life history traits in prey species is largely unknown.

In most coral reef ecosystems, quantifying the relative impacts of different fish predators on their prey has been problematic. Large mesopredatory and apex species such as sharks that prey on other fishes are highly mobile and interactions with their prey occur unpredictably and often in a transitory manner^16,17^. Furthermore, the presence of an observer may also be perceived by prey fishes as a threat, confounding responses to predators and reducing the likelihood of predatory interactions^18^. To circumvent these issues, researchers have used life-sized models of predators to evoke antipredator responses in reef fishes. This is typically combined with the use of remote cameras to avoid disturbance from an on-site observer. To date, these studies have used only static models^17–20^, which realistically mimic ambush predators such as coral trout and groupers (Serranidae^18,20^), but do not imitate the behaviour of many of the large predators in the reef systems, such as sharks (but see^19^). These large animals are highly mobile in both horizontal and vertical planes^21,22^ and move over extensive home ranges^14,23^. For this reason, a static model may not offer a realistic simulation of predatory threat because, unlike a live shark, the model is continually present within the experimental arena and thus might offer a greater degree of threat to prey species. Additionally, because the model is inactive, prey may habituate to its presence^18,24^. One effective solution to this issue is the use of animated models that can provide a predator threat in a more realistic manner to prey species. Such an approach has been used in the study of bird ecology, where researchers have animated models of hawks and other apex predators to examine the anti-predatory responses of prey^24,25^.

The use of animated models of fish predators offers the opportunity to compare impacts of fish and human predators in a single experiment. Here, we examine the behavioural responses of mesopredatory reef fishes to the presence of shark models and human snorkelers in a coral reef ecosystem. Spearfishing by snorkelers is an important means of harvesting species for recreational, artisanal and small-scale fisheries in these environments. Unlike many other types of fishing, this activity is known to have predictable impacts on the behaviour of reef fishes that increase their responses to reduce capture risk^12,26–29^. For example, flight initiation distance (FID; defined as the distance prey allow a potential threat to approach before fleeing^30^) is much greater in areas where fish are frequently targeted by spearfishers than in places where spearfishing does not occur^26,29,31^.

Mesopredators, including lethrinids, haemulids, lutjanids and serranids (emperors, grunts, snappers and groupers), are relatively large reef fishes that are an important target for spearfishing in coral reef systems and at the same time show threat responses to sharks as a potential predator and/or a lethal competitor^17,32^. We used an experimental approach to compare the threat response of these reef fishes to animated models of sharks and to the presence of snorkelers. We used standardized metrics (including FID) to quantify the response of reef fishes to these threat treatments compared to animated models that did not pose a predation threat (sea turtles and two object controls). As prey individuals of large body sizes tend to display the greatest responses to predatory threat^20,26,33,34^, we predicted that FID would be greater for larger than smaller mesopredators in the presence of both animated models of sharks and snorkelers. Additionally, we used the difference of swimming speed prior to treatment approach and during flight response to calculate speed of flight as a measure of threat response to the different treatments. We predicted a higher difference in swimming speed during the approach of threatening treatments compared to the approach of the non-threatening animated models and controls and expected a greater difference in swimming speed for larger mesopredatory fishes during the two threatening treatments.

## Materials and methods

### Study site and experimental design

**Figure 1:**
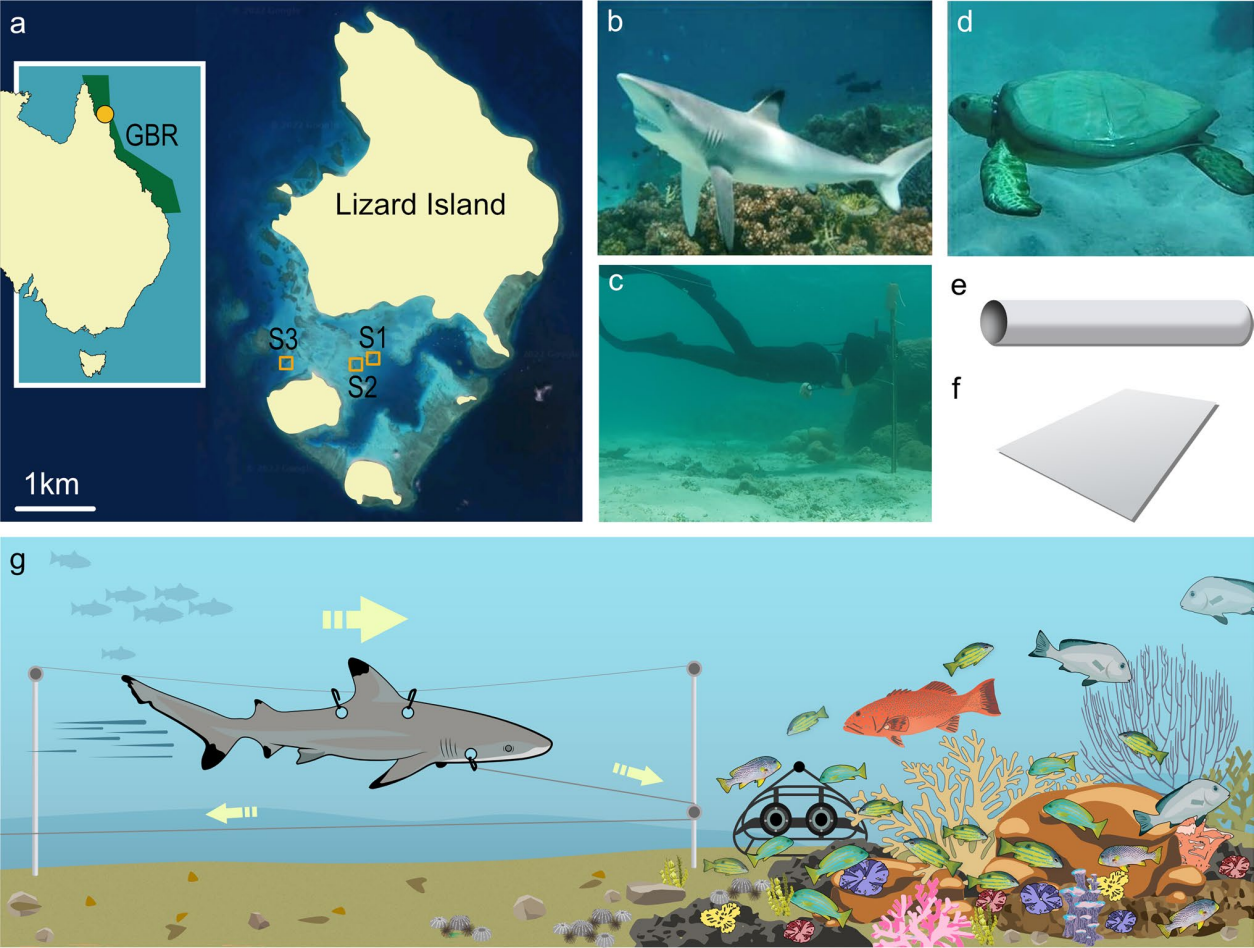
(**a**) Sample sites () located on the fringing reefs surrounding Lizard Island, located in the northern section of the Great Barrier Reef, Australia (map courtesy of Ooid Scientific). Photograph of a (**b**) blacktip reef shark model, *Carcharhinus melanopterus* (Simpson S.D.), (**c**) photograph of a snorkeler (180 cm TL, Asunsolor-Rivera A.) (**d**) photograph of a green sea turtle model (non-threatening model, Lester E.), (**e**) PVC pipe (control object, courtesy of Ooid Scientific) and (**f**) transparent Perspex (negative control object, courtesy of Ooid Scientific). (**g**) Shark model attached to pulley system and remote underwater video system (RUVS) placed near the edge of the coral bommie. Large arrow represents the shark model moving towards the coral bommie. Small arrows represent the traveling direction of the line in the pulley system (**g** courtesy of Ooid Scientific).

The study was conducted in November and December of 2017 on the fringing reefs surrounding Lizard Island (− 14.668589°, 145.463777°) a no-take zone or IUCN II Marine Park, in the northern Great Barrier Reef (GBR), 30 km from the mainland (Fig. 1a).

The experiment was conducted at three sites within the lagoon (Fig. 1a). Sites were separated by a minimum of 100 m and were selected following surveys of the reefs that recorded the presence of schools of mesopredatory reef fishes (haemulids, serranids, and lutjanids) that were resident on large coral heads located on the reef edge during the daytime.

At each site, star pickets were hammered into the sand at the edge of the reef immediately adjacent to a large coral head where mesopredators congregated predictably. A second picket was placed 10 m out onto the sandflat perpendicular to the reef edge. A length of fishing line was attached between the top of these pairs of star pickets to construct a pulley system (Fig. 1g). A small dive weight was attached to the end of the fishing line on the second picket to provide a hand hold to draw the line along the pulley system and to keep the lines in place when not in use. A total of six pulley systems were constructed with a minimum distance of 25 m separating two adjacent systems within each site, but only five of the six were used in the experiment as mesopredatory fishes were not present reliably at one pulley system.

To experimentally examine the behavioural response of mesopredators to predation threat, we used five treatments (two threatening, and three controls) during each trial. These treatments consisted of a life-sized (170 cm total length; TL) fiberglass taxidermic casting of a blacktip reef shark (*Carcharhinus melanopterus*) (Fig. 1b; as per^18^), which is a locally abundant predator on the Great Barrier Reef^35^. A snorkeler was used as the second threatening treatment (~ 180 cm TL; Fig. 1c). The first control was a model of a green turtle (*Chelonia mydas;* 65 cm TL; Fig. 1d), which presents no threat to mesopredatory fishes as the species feeds on seagrass. A white PVC tube (81 cm TL, 9 cm diameter; Fig. 1e) was used as a shape control, which examined the possibility that mesopredatory fishes may react to the presence of a novel object (“neophobia”; as per^18^). Finally, a piece of transparent Perspex (81 cm TL; Fig. 1f) was used to control for the effect of sound and water movement created by the action of the pulley system.

These models were attached to the fishing line near the top of the star pickets via hooks on the top of the model and another hook on the front of the model was attached to a clip on the pulley system, which allowed the model to be drawn along the pulley line. To move the model, the dive weight at the end of the line was retrieved by a snorkeler who swam away from the reef at a constant pace of 28–31 s per 10 m distance, which pulled the model towards the reef and associated mesopredators. For the snorkeler treatment, the snorkeler followed the pulley system and swam towards the reef at the same depth as the models and objects attached to the lines.

The experimental trials were recorded using unbaited Remote Underwater Stereo-Videos Systems (RUVS), which consisted of two cameras (GoPro HERO3 Silver) mounted 0.7 m apart on a steel frame, facing 8° inwards to achieve the optimal field of view and calibrated before and after measurements, as per^36^. Trials were conducted between 08:00 and 17:00 h to avoid crepuscular behaviours.

Deployments of treatments were randomised with at least 20 min between deployments. Three replicate trials were conducted per treatment at each of the five pulley systems (15 trials per treatment), resulting in a total of 75 videos. A minimum wait of 24 h separated any treatment replicates within a pulley system. One replicate trial was discarded due to the presence of a live blacktip reef shark appearing in the video.

### Video analysis

The video footage was analysed using EventMeasure software (SeaGIS Pty Ltd 2011). Mesopredators on coral heads were mostly representatives of the reef fish families Haemulidae, Serranidae and Lutjanidae (see Table S1, Supplementary Information). Individuals of these families were identified to species in the videos. Principal component analysis suggested that there was no systematic variation in species composition among treatments or controls (Fig. S1 Supplementary Information). The following measurements were extracted from each video: flight initiation distance (FID), total length of each fish (body length), vertical height from benthos (VHB; the vertical distance between the mesopredator and the benthos), and number of fish (individual or school; individuals of the same species swimming in a coordinated manner) at start of trial, and speed of reaction or speed of flight (Table 1). These measurements were estimated for all mesopredatory fishes within six meters of the cameras (within range of water visibility, as per^37^) of the RUVS to ensure accuracy of measurements of lengths or distances^38^. For each video the reactions of individual fishes to the approach of the treatments were categorized into four responses: C-turn, flight, hide, and no response, with the latter indicating that the fish did not move or change its position at any point during approach of the object on the pulley system or the snorkeler (Table 1).

**Table 1:** Variables measured and type of behavioral response (*) during video analysis with corresponding description.

Measurement	Description
FID	Measured at the moment of first reaction, from the most forward point of each treatment (i.e. the middle of the snout in the shark model) to the tail or eye of each fish, depending on which way the fish was facing. In some cases fish became aware and wary of the treatment approaching, known as distance of first alert^82^, sometimes changing the direction they were facing prior to flight, but FID was always measured the instant the fish began to flee
Total length of prey fish (body length)	The length was measured when the fish was fully extended parallel and as close to the camera as possible
Treatment size	The size of treatment was included as an explanatory variable where: Shark: 170 cm, Snorkeler: 180 cm, Turtle: 65 cm, Pipe: 81 cm and Perspex: 81 cm This variable was included in the model to account for the difference in size between the threat treatments and the controls
No. of fish	Whether the fish was swimming as an individual or within a school. A school was defined as three or more fish swimming closely together and synchronizing movements. To analyze the escape response of a school, three individuals were haphazardly selected for measurement and the average value of these measurements used to represent the behaviour of the school
Vertical height from benthos (VHB)	Measured from the lowest point of the fish to the nearest benthos below the fish. This measurement was taken three times: nine, six, and two seconds prior to FID. If the fish was not within frame for those nine seconds prior to FID the measurements were taken three, two and one second prior to FID. The mean of the three measurements was included in statistical models
Speed of flight	The difference between mean swimming speed of a fish prior to approach of the treatment and speed after the arrival of the treatment. This was calculated by measuring the distance of movement every three seconds (three, six and nine seconds) prior to treatment approach and dividing it by the time (three seconds). The swimming speed of flight was calculated using position coordinates to measure the distance^40^ from the flight response to the time when the fish was no longer visible or hiding
C-turn*	A 90°–180° turn before swimming away out of the field of view of camera
Flight*	A fish swam away from the treatment and out of the field of view of the camera in the direction it was already facing
Hide*	Swimming into a crevice or hole in the reef
No response*	No change in behaviour during the approach of the treatment

To measure the speed of flight, we used EventMeasure software to calculate the difference in swimming speed before and after reaction to the approach of the treatment for each fish in the field of view. Speed prior to flight was calculated for each fish by measuring the distance travelled by placing position coordinates^39^ on the eye^40^ or tail of the fish (perspective dependent) at three, six and nine seconds prior to flight. A small piece of Blu Tack was placed on the screen to mark the position coordinate and the distance was measured between two consecutive position coordinates^40^. These distance measurements were converted to speed using the Eq. (1):

1 $$\mathrm{S}=\mathrm{d}/\mathrm{t}$$

where $\mathrm{S}$ = Speed, $\mathrm{d}$ = distance in meters and $\mathrm{t}$ = time in seconds.

For most individuals, distance could be measured as a straight line and divided by 3 s. However, some individuals turned or changed swimming direction, and in these cases the position coordinate was placed at the turning point and the swimming speed was calculated as the distance covered by the mesopredator and the time elapsed on the video. We calculated a minimum of three separate measurements of swimming speed for each individual fish, which were then used to calculate mean swimming speed prior to flight. A similar process was then used to calculate speed of flight, where position coordinates were used to calculate distance and divided by swimming time. The difference between speed of flight and mean swimming speed prior to flight was used as a response variable in statistical models.

Only fish swimming within a range of angles from 90° to 70° to the camera were included in the speed of flight analysis. If a fish was swimming outside this range (e.g., directly away from the camera), it was excluded from the analysis as distance swum could not be calculated accurately^37^.

### Data analysis

Generalized additive mixed models (GAMMs) with a full-subsets information theoretic approach was used to examine the influence of treatment, treatment size, fish body length, fish genus and number of fish on FID and difference in swimming speed of fish before and after flight was initiated. The effect of VHB was only examined on FID, as measurement of VHB after flight had begun was logistically challenging to standardize. The five treatments (categorical; shark, snorkeler, turtle, pipe and Perspex), genus of mesopredator (categorical: *Lutjanus*, *Diagramma*, *Plectorhinchus* and *Plectropomus*), body length (continuous), and number of fish (categorical; school or individual) were included as fixed factors. Body length was log_10_ transformed for all analyses to ensure that the data were normally distributed (See Figs. S2–S9 Supplementary Information). Pulley system was nested within site and included in each model as a random factor.

The package *FSSgam*^41^ was used to construct, fit and compare all possible models with a maximum of three predictor variables. This limitation of predictor variables avoided over-fitting and ensured the models remained ecologically interpretable. Models were compared using Akaike’s Information Criterion corrected for small sample size (AICc^42^) and AICc weights (wAIC^43^) were used for model selection. Models containing variables with correlations > 0.28 were excluded from the analysis to eliminate strong collinearity (as per the recommendations of Graham)^44^. Models with AICc values that differ by less than two units show weak evidence for favouring one over the other^43^. For this reason, the most parsimonious model was considered the one with the fewest variables and within two AICc units of the lowest AICc value^45^. The wAICc were used to assist in the interpretation of the best models. The summed wAICc across all subsets of models was used to obtain the relative importance of each variable^45^. Models were fitted to a Gaussian distribution and identity-link function, as all response data were approximately normally distributed. All analyses and graphical representations were conducted using R^46^ with the packages *dplyr*^47^, *ggplot2*^48^ and *Patchwork*^49^.

In addition to the information-theoretic approach, a univariate permutational analysis of variance (PERMANOVA) was used to independently test for significance (95% confidence) and appropriate pair-wise comparison (as per^50^) for the most parsimonious models for both FID and speed of flight. The PERMANOVA was analysed using PRIMER v6 and the add-on package PERMANOVA+; with 9999 permutations^51^. For this analysis four fixed factors were included: treatment (five levels: shark, snorkeler, turtle, pipe and Perspex), genus of mesopredator (four levels: *Lutjanus*, *Diagramma*, *Plectorhinchus* and *Plectropomus*), body length (continuous) and number of fish (two levels: school or individual) and one random factor: pulley system (five levels), which was nested within site.

### Ethics declarations

Permits/ethical approval–Animal ethics approval was obtained from James Cook University under approval numbers A2080 and A2350. Experiments were conducted at Lizard Island Research Station under permit number G12/35236.1 issued by the Great Barrier Reef Marine Park Authority, permit number 191960 issued by Department of Agriculture, Fisheries and Forestry, Queensland Government, Australia. All applicable institutional and/or national guidelines for the care and use of animals were followed. All methods were reported in accordance with ARRIVE guidelines for the reporting of animal experiments.

All experimental protocols were approved by James Cook University (ethics number A2350) and were conducted within University guidelines. Informed consent was obtained from all human subjects.

## Results

### Type of escape response in mesopredatory fish

**Figure 2:**
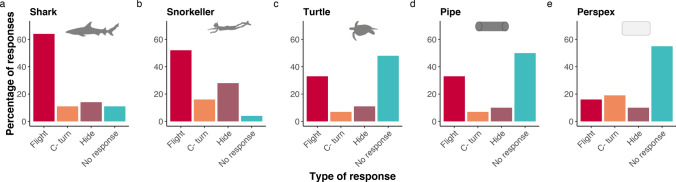
Type of escape response in proportion to treatment. Three different types of escape responses were identified: Flight, C-turn and Hide. The No response reaction indicates the fish did not change position as the treatment approached.

Overall, 90% of mesopredatory fishes displayed an escape response (c-turn, flight or hide) when approached by the shark model, and 96% displayed an escape response to a snorkeler. In contrast, the turtle, pipe and Perspex treatments had very high proportions of fish displaying no response (44%, 50% and 55% respectively; Fig. 2).

### Models and variable importance scores

**Figure 3:**
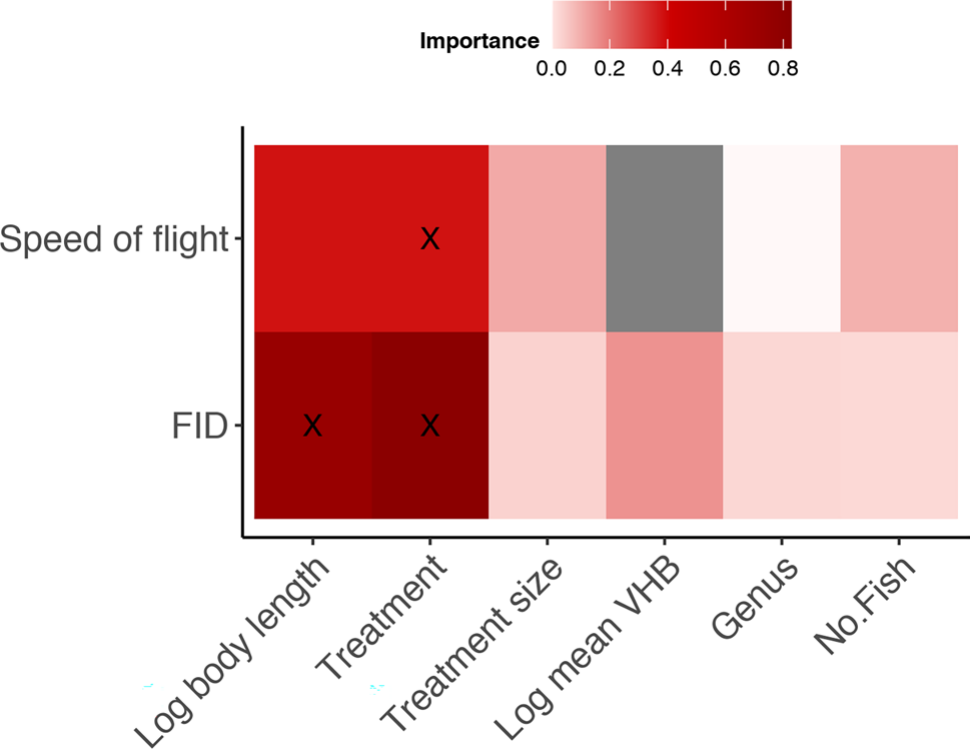
Variable importance scores from full subset GAMM analyses predicting flight initiation distance (FID) and speed of flight according to prey body length, treatment, treatment size, vertical height from benthos (VHB), genus of mesopredator (Genus) and number of fish (individual or school). X indicates the variables within the most parsimonious model. Grey indicates the variable was not included in the model.

**Figure 4:**
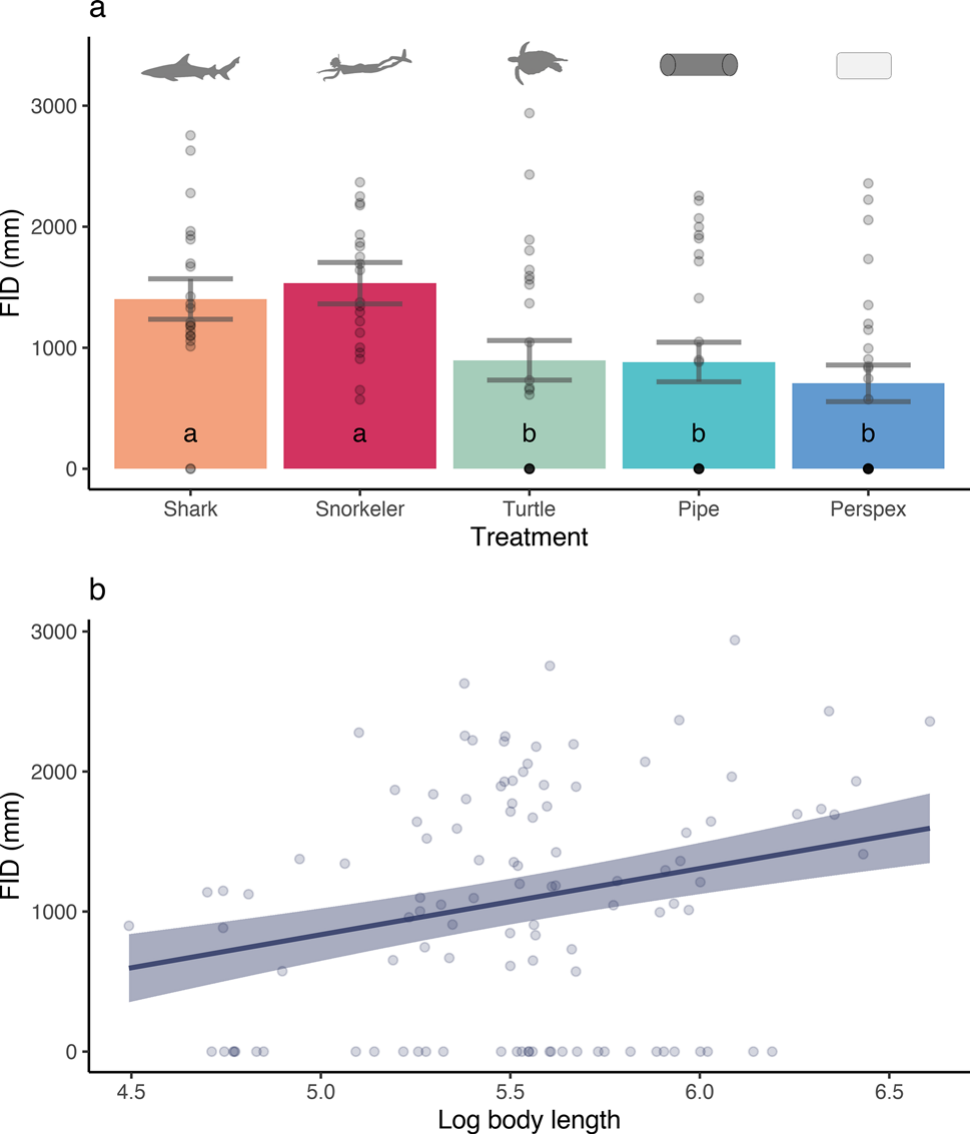
(**a**) Average (± SE) flight initiation distance (FID) for each treatment. Points indicate distribution of raw data. The results of pairwise comparisons are indicated by an alphabetic character. (**b**) Flight initiation distance (FID) relative to the body length of mesopredatory fishes. The solid line indicates the estimated smoothing curve and the shaded area indicates the ± SE of the estimate. Points show distribution of raw data.

The most parsimonious model for flight initiation distance (FID) included treatment and body length, which collectively explained 21% of the variation (Table 2). The importance scores indicate that VHB was also relatively important in predicting FID (Fig. 3). Model predictions showed that FID was larger in the presence of the threatening treatments (shark model: 1400 mm; snorkeler: 1500 mm), and lower in the presence of the non-threatening model and the novel object (turtle: 900 mm; pipe: 900 mm). Average FID was lowest for the Perspex treatment (600 mm), but not significantly different from the turtle and pipe treatments (Fig. 4a). Additionally, FID was positively correlated with the log_10_ of body length (Fig. 4b).

**Table 2:** Best generalized additive mixed models (GAMMs) for predicting FID and speed of flight in reef mesopredatory fish from full subset analyses.

Dependent variable	Best fit models (ordered by parsimony)	ΔAICc	ΔBIC	ωAICc	ωBIC	R^2^	EDF
FID	Treatment + log. Body length	1797.86	1815.74	0.785	0.435	0.21	7
Speed of Flight	Treatment	− 99.57	− 82.13	0.595	0.12	0.18	6
	Treatment + log. Body length	− 100.16	− 82.36	0	0.134	0.18	7
	Log. Body length* Treatment + Treatment	− 99.05	− 72.04	1.109	0.001	0.24	11

This result was corroborated by a PERMANOVA, which found significant effects of treatment (Pseudo-F = 6.91, df = 4, p < 0.001) and body length on FID (Pseudo-F = 5.25 df = 3, p =  < 0.01). Pairwise comparisons indicated a significant effect of body size on FID for the smallest size class, with a larger FID in mesopredatory fishes > 200 mm (t = 4.13, df = 43, p < 0.001) than individuals ≤ 200 mm in length (See Fig. S10 Supplementary Information). Although there was no significant difference in FID between the smallest (≤ 200 mm) and the larger size classes (≥ 260 mm), there was a trend in increasing FID with larger body sizes. Pairwise comparisons of treatments showed that there was no difference in FID of mesopredators between the shark model and snorkeler treatments (t = 1.00, df = 28, p > 0.05). There was a significant increase in FID in response to the shark compared to Perspex (t = 3.22, df = 34, p < 0.01), pipe (t = 2.54, df = 29, p = 0.016) and turtle (t = 2.47, df = 30, p = 0.01) treatments. FID also increased significantly in the snorkeler treatment compared to Perspex (t = 5.18, df = 34, p < 0.001), pipe (t = 3.27, df = 29, p =  < 0.01) and turtle (t = 3.38, df = 30, p = 0.001) treatments.

**Figure 5:**
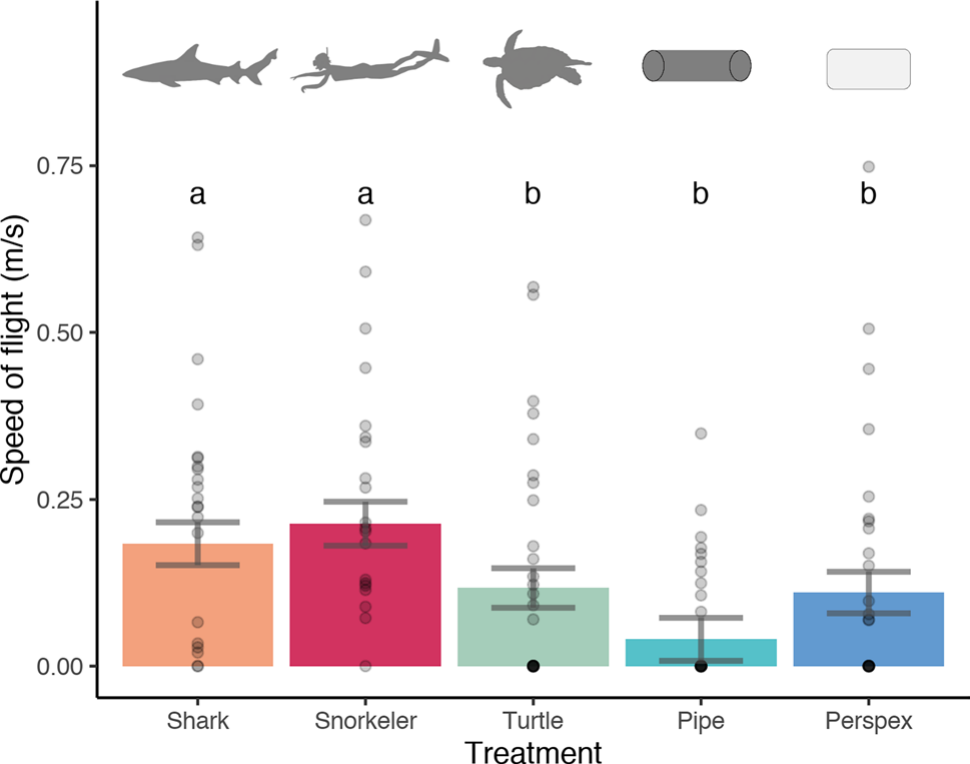
Average speed of flight (± SE) for each treatment. Points show distribution of raw data. The results of pairwise comparisons are indicated by an alphabetic character.

The most parsimonious model of speed of flight included only treatment, which explained 18% of the variation (Table 2). Body length and number of fish (individual or school) were also relatively important in predicting speed of flight, but were not included in the most parsimonious model (Fig. 3). Speed of flight was higher in the shark (0.19 ± 0.03 m/s) and snorkeler (0.21 ± 0.03 m/s) than the non-threatening treatments (turtle: 0.11 ± 0.03 m/s, pipe: 0.04 ± 0.03 m/s, and Perspex: 0.11 ± 0.03 m/s) (Fig. 5).

This result was corroborated by a PERMANOVA, which found significant effects of treatment (Pseudo-F = 4.66, df = 4, p = 0.001). Pairwise comparisons found no difference in the speed of flight of mesopredators in shark and snorkeler treatments (t = 0.67, df = 28, p > 0.05). There was, however, a significant increase in the speed of flight in the shark compared to the pipe treatment (t = 3.71, df = 29, p < 0.001), but no difference between shark and turtle (t = 1.94, df = 33, p > 0.05,) or shark and Perspex (t = 1.12, df = 31, p > 0.05) treatments. The speed of flight differed significantly between the snorkeler and turtle (t = 2.80, df = 33, p < 0.01), snorkeler and pipe (t = 4.13, df = 28, p < 0.001) and snorkeler and Perspex (t = 2.15, df = 31, p < 0.05) treatments.

## Discussion

Our study found that large mesopredatory reef fishes responded to the approach of non-human and human threats (animated shark model and snorkeler) by displaying greater flight initiation distances (FIDs) and faster speeds of flight compared to responses to non-threatening models (turtle) or object controls. The approach of the model shark and snorkeler elicited similar FID and speed of flight reactions, although it was not clear whether mesopredatory fishes might regard these threats as potential predators or lethal competitors.

Lethal competition or intraguild predation, defined as the killing and consumption of a potential competitor^52,53^, can occur across the guild of large predatory reef fishes. Reef sharks, such as the blacktip, are known to feed on large predatory teleosts^54^, but are also known to compete with these fishes for smaller prey^55,56^; thus the escape response triggered by the shark model could be interpreted as an anti-predatory behaviour and/or a response to a potentially lethal competitor. Regardless of the motivation for the escape response, our key finding is that the approach of a threat elicited a flight response in mesopredatory fishes and one that may plausibly result in an individual forgoing the opportunity to forage, interact or compete with other individuals of the same or other species in the vicinity. This adds to the evidence that the presence of larger predators such as reef sharks and humans may ultimately create risk effects within reef fish populations.

Body length was positively correlated with FID in mesopredatory fishes, as has also been seen in birds^57,58^. Earlier studies that focused on spearfishers as a threat have also found a similar correlation with size in many^26,29^, but not all coral reef fishes^31^. Gotanda et al.^33^ attributed this positive relationship to the lower risk-taking associated with the higher reproductive value of larger fish, consistent with predictions of the asset-protection principle, which states that as reproductive value increases, risk-taking should decrease^59^. Another (non-exclusive) possibility is that larger fish are older and have more experience with potential predators, thus are more wary. There are many field studies that show that prey can learn from earlier attempts at predation directed towards them and their conspecifics^60–63^. Additionally, larger mesopredatory fishes are more likely to be competitors with reef sharks, so may be under greater threat as they increase in size.

Other studies have concluded that the increased FIDs with size could be attributed to higher spearfishing pressure on larger fishes^26,64^. However, our experiment took place in a no-take zone, where there is theoretically zero fishing pressure, and all fish are protected from spearfishing regardless of their size. This implies that other factors might explain the greater FIDs seen in larger fish. Interestingly, a recent meta-analysis conducted on FID studies failed to detect any effect of habitat protection status as an explanation of the body length-FID relationship^29^. Although our experiment was conducted immediately adjacent to coral heads, the proximity of shelter can also influence FID^65^. For example, Lester et al.^17^ found that mesopredatory fishes took longer to feed in the presence of a shark model at greater distances from the shelter of a patch reef. Larger fish may require relatively large crevices and holes in which to shelter from predators, which may force them to move earlier in response to a threat than smaller species if these refuges are rare on the reef. It is notable that the FIDs we recorded in this study were only a third of the distances reported for large mesopredators on the reef slopes of coral reefs with only moderate complexity^26^. This implies that reef slopes may offer less shelter than the coral heads in the lagoon and/or that there may be higher predation pressure on large mesopredators on the reef slope. Certainly, relative densities of sharks as measured by BRUVS and underwater visual surveys are higher in the reef slope habitat than the shallow lagoon or backreef^35,66^. Rigorous comparisons of FIDs across habitats will require both empirical measures of habitat complexity and of the abundance and distribution of shark populations.

The ability of prey to detect an approaching threat can influence the level of wariness and willingness to take risks^67,68^. Visual acuity is expected to increase in larger fish^69^, allowing them to detect the approaching threat before smaller fishes. In addition, larger fishes may have better sensory systems to detect cues that prompt anti-predatory behaviour^70^, such as smell^71^, electrosensory and pressure fields^72^, alarm calls from other conspecifics and predator calls^4,73^. Future experiments that involve other sensory systems could provide a more comprehensive approach to threat detection and the associated anti-threat behaviours of mesopredatory fishes.

We found that speed of flight increased with body size of mesopredators. These results contrast to those of Miller et al.^34^, who found that smaller fish had faster escape speeds, as is also the case with size in lizards^74–76^. These earlier studies suggested that their results were due to trait compensation, whereby individuals with a trait that made them more vulnerable to predation had stronger antipredator behaviours than those that lacked this trait. In our study, the positive relationship between size and escape speeds may be an example of co-specialisation of anti-predator behaviours^77^, similar to other fishes such as sturgeon (*Acipenser fulvescens*) where larger body size is also positively correlated with stronger and more sustained escape responses^78^.

It is important to note that our object controls were smaller than either the model reef shark or the snorkeler. Although it is possible that the differences in reaction between our controls and threatening treatments were a response only to relative differences in sizes of the objects approaching the reef, size was not included within the most parsimonious model in our analysis. Similarly, an earlier study^18^ found that reef fishes showed the same response to the threat of a model of a large coral trout that was 78 cm in length and a model of a black tip reef shark of 170 cm in length, despite the over two-fold differences in relative size of these predators. In this case, the control treatment (a pipe) was the same size as the large coral trout. Such differences in relative sizes of predators have also been found to have little effect on anti-predator behaviour of prey of fishes in other experimental systems^79^, suggesting the effect of predator size in evoking antipredator behaviours may not be as significant as predator identity and other visual cues^61^. Fish are well known to recognize features, such as body shape, presence and position of eyes and shape of mouth to identify potential predators^80^, although disentangling the effect of size and visual cues in prey recognition has proven challenging to date.

Finally, our results have implications for researchers seeking to survey reef communities or monitor behaviour, since they show that the presence of an observer could induce behaviours in reef fishes that seek to reduce potential predatory threat. This might provide an explanation as to why techniques such as underwater visual census often underestimate densities of the larger, mobile reef fishes within coral reef ecosystems^81^.

## Supplementary Information

Supplementary Information.

## Data Availability

The datasets used and/or analysed during the current study are available from the corresponding author (Andrea Asunsolo-Rivera) on reasonable request and will be provided via a dryad repository.
