# Supplementary material for: Behaviour of mesopredatory coral reef fishes in response to threats from sharks and humans
Source: Sci Rep. 2023 Apr 25;13:6714. doi: 10.1038/s41598-023-33415-5 (PMC10130163; doi:10.1038/s41598-023-33415-5)
Supplement: Supplementary file 1 — Supplementary Information. [file 41598_2023_33415_MOESM1_ESM.docx]

**Supporting Information**

**Species composition**

The escape responses of 111 fishes were recorded across trials. A total of 10 species and four genera were included with the most common the haemulids, *Diagramma pictum labiosum* (n=28) and *Plectorhinchus chrysotaenia* (n=20) and the lutjanid, *Lutjanus fulviflamma* (n=26) (Table S1).

Table S1. Identity and abundance of mesopredatory fishes recorded during videos.

| Species | Total number |
| --- | --- |

| *Diagramma pictum labiosum* | 28 |
| --- | --- |
| *Lutjanus bohar* | 3 |
| *Lutjanus fulviflamma* | 26 |
| *Lutjanus sp* | 14 |
| *Plectorhinchus chrysotaenia* | 20 |
| *Plectropomus leopardus* | 5 |
| *Lutjanus carponotatus* | 5 |
| *Lutjanus fulvus* | 3 |
| *Plectorhinchus albovittatus* | 2 |
| *Plectorhinchus lineatus* | 5 |

A principal coordinates analysis followed by a PERMANOVA was used to test for differences in the species composition per treatment, using PRIMER v6 and the add-on package PERMANOVA+; with 9999 permutations (Anderson et al., 2008). The experimental design for this analysis consisted of two fixed factors: species (10 levels) and treatment (five levels: shark, turtle, pipe, perspex and snorkeler).

No significant difference was found in species composition of mesopredatory fishes across model replicates (p-value = 0.7). A biplot of principal components suggested that there was no systematic variation in species composition among treatments or controls (Figure S1).


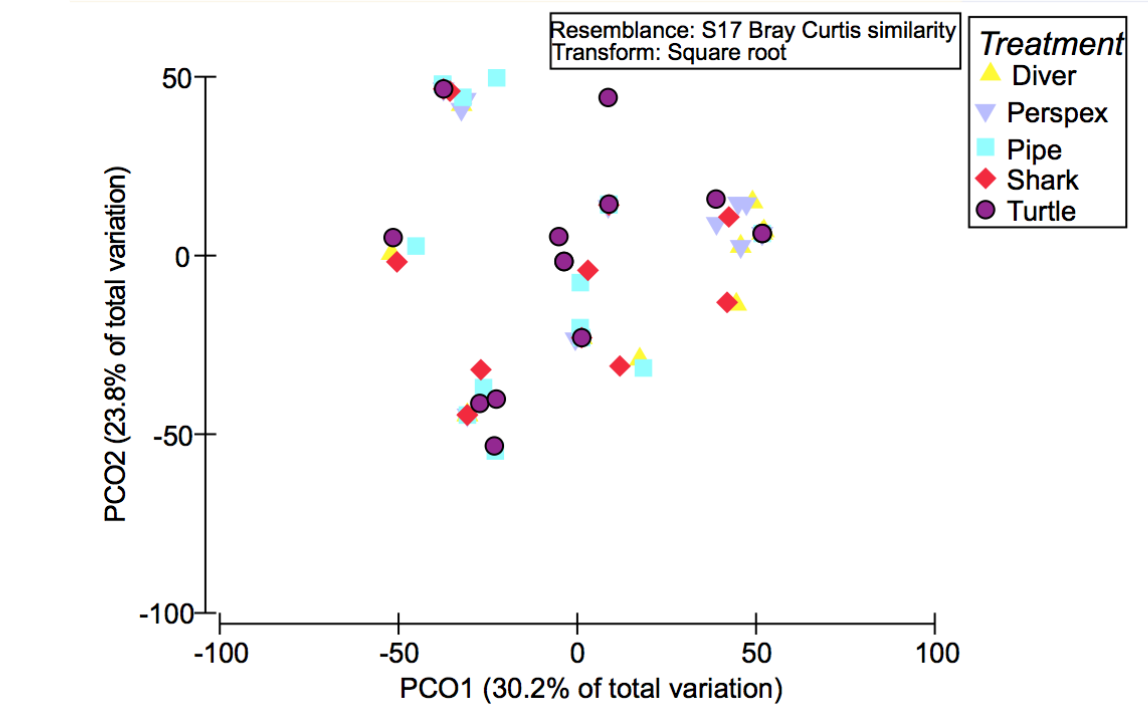


Figure S1. Principal coordinates analysis of the fish composition by treatment.

After conducting the Generalized additive mixed models (GAMMs) we used the model diagnostics using the gam.check function to test for normality and confirm we could not find any deviations in the residuals for the Flight initiation (FID) model (Figures S2 - S5) and the speed of flight model (Figures S6 – S9).

Figure S2. Gam check diagnostic model of theoretical quantiles for FID.

Figure S3. Gam check diagnostic model of residuals vs. linear predictors for FID.

Figure S4. Gam check Histogram of residuals for FID model.

Figure S5. Gam check diagnostic model of response vs. fitted values for FID.

Figure S6. Gam check diagnostic model of theoretical quantiles for speed of flight.

Figure S7. Gam check diagnostic model of residuals vs. linear predictors for speed of flight.

Figure S8. Gam check Histogram of residuals for speed of flight model.

Figure S9. Gam check diagnostic model of response vs. fitted values for speed of flight.


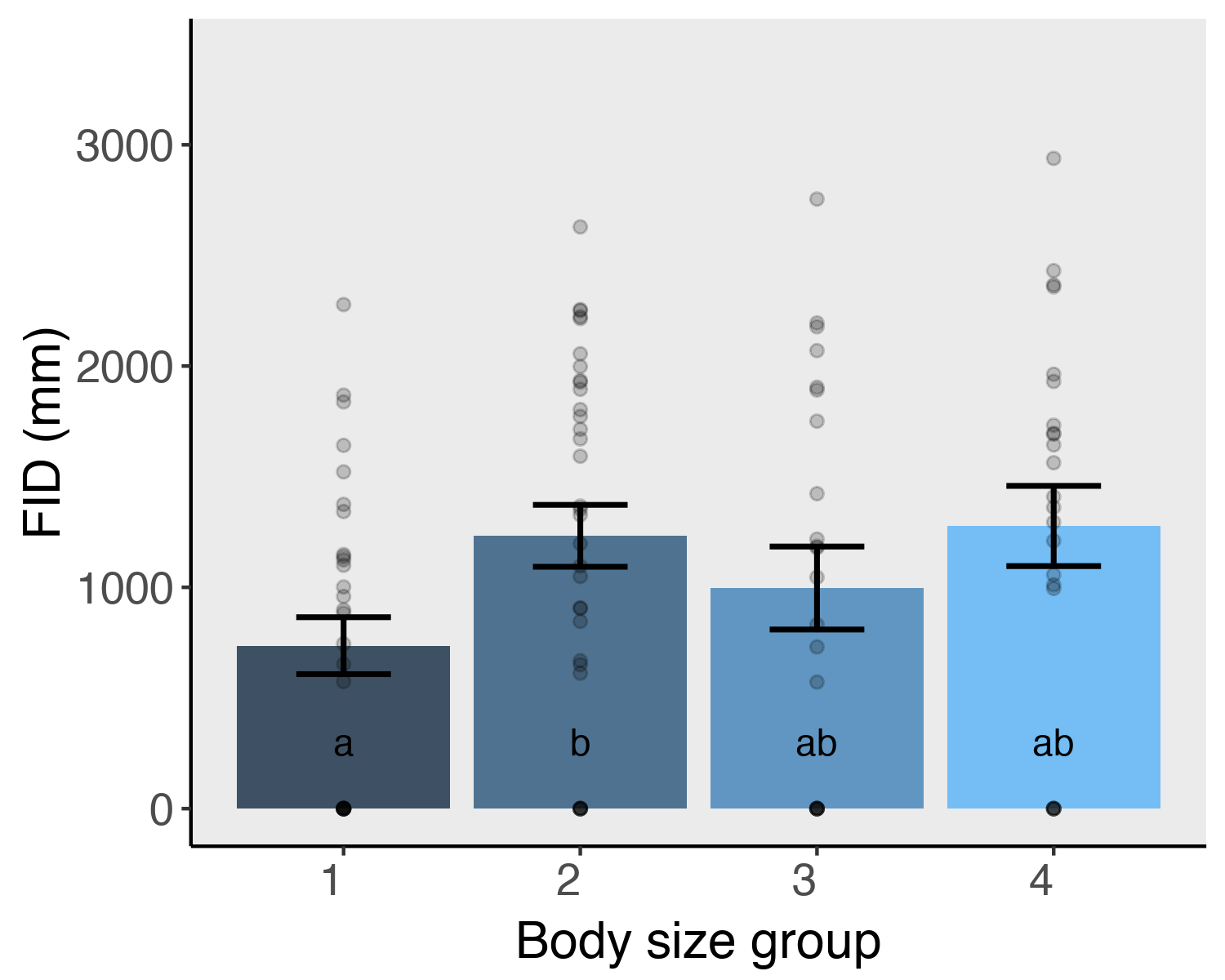


Figure S10. Average (±SE) flight initiation distance (FID) per body length group. Points indicate distribution of raw data. Body length group 1 = 88 – 199 mm, group 2 = 200 – 259 mm, group 3 = 260 – 359 mm and group 4 = 360 – 739 mm. The results of pairwise comparisons are indicated by an alphabetic character.
